# Impact of β-Carotene Enrichment on Carotenoid Composition and Gene Expression in *Artemia* Metanauplii

**DOI:** 10.3390/metabo14120676

**Published:** 2024-12-03

**Authors:** Weilong Wang, Zhuojun Ma, Weiquan Li, Yucai Xue, Amina S. Moss, Meiqin Wu

**Affiliations:** 1 Building of China-ASEAN Belt and Road Joint Laboratory on Mariculture Technology, Shanghai 201306, China; wangweilong@shou.edu.cn (W.W.); m220100242@st.shou.edu.cn (Z.M.); 2211705@st.shou.edu.cn (W.L.); d220100029@st.shou.edu.cn (Y.X.); 2 Centre for Research on Environmental Ecology and Fish Nutrition of the Ministry of Agriculture, Shanghai 201306, China; 3 National Demonstration Center for Experimental Fisheries Science Education, Shanghai Ocean University, Shanghai 201308, China; 4 Institute of Aquaculture, University of Stirling, Stirling FK9 4LA, UK; amina.moss@stir.ac.uk; 5 College of Oceanography and Ecological Science, Shanghai Ocean University, Shanghai 201306, China

**Keywords:** *Artemia*, β-carotene, enrichment, transcriptome analysis, metabolic pathways

## Abstract

Background: Carotenoids play essential nutritional and physiological roles in aquatic animals. Since aquatic species cannot synthesize carotenoids de novo, they must obtain these compounds from their diet to meet the physiological and adaptive requirements needed in specific aquaculture stages and conditions. Carotenoid supplementation in *Artemia* represents a promising strategy to enhance pigmentation, health, and growth in aquaculture species, particularly in larvae and other early developmental stages. Methods: In this study, a β-carotene enrichment process was applied to *Artemia* metanauplii to investigate the biological fate and potential effects of β-carotene. Results: The results indicated significant β-carotene uptake by *Artemia*, with peak levels observed at 12 h. Alongside β-carotene, two xanthophylls—canthaxanthin and echinenone—were detected in *Artemia*, each exhibiting distinct patterns during the enrichment and subsequent depletion phases. The transcriptome analysis identified 2705 differentially expressed genes (DEGs), offering valuable insights into gene functions associated with carotenoid absorption, metabolism, and antioxidant mechanisms. The findings suggest that β-carotene enrichment enhances metabolic activity and energy pathways, supporting the physiological functions of *Artemia*. Notably, unlike other crustaceans, *Artemia* lack certain enzymes necessary for converting β-carotene into astaxanthin, restricting them to producing keto-carotenoids like canthaxanthin. Furthermore, the study highlights the upregulation of genes encoding lipid transport proteins, such as CD36 and ABC transporters, which may contribute to carotenoid absorption in *Artemia*. Additional functional insights are provided by the gene BCO2, which regulates pigmentation by preventing excessive carotenoid accumulation, along with ketolase and hydroxylase enzymes in carotenoid metabolic pathways. Conclusions: This research advances our understanding of carotenoid metabolism in crustaceans, with potential implications for aquaculture nutrition and feed formulation.

## 1. Introduction

Carotenoids represent a group of conjugated, double-bonded, long-chain terpenoid compounds that are widely distributed in the flesh, skin, and exoskeleton of aquatic animals [1]. The pigmentation and immune function of aquatic animals are closely associated with the composition and concentration of carotenoids within their bodies [2]. In natural ecosystems, carotenoids are synthesized by microorganisms and subsequently accumulate through the food chain, involving zooplankton, crustaceans, and fish, which themselves lack the capability for the de novo synthesis of carotenoids [3]. However, intensive aquaculture practices have disrupted these natural communities, resulting in a scarcity of effective pigment sources. This disruption is a key factor leading to carotenoid deficiencies in cultured species [4]. To address this issue, carotenoid supplementation in feed has become an important strategy in aquaculture to improve coloration, enhance taste, boost disease resistance, and support reproductive performance [2].

Despite advancements in formulated diets, rearing early-stage aquatic animals remains heavily dependent on live feed [5]. Among live feeds, the newly hatched nauplii, metanauplii, and adults of the micro-crustacean *Artemia* spp. are widely utilized for marine larval rearing, owing to their availability and acceptance across diverse species [6,7]. Traditionally, *Artemia* has been considered an excellent larval live feed for larvae, primarily due to its provision of digestible protein [6]. Furthermore, *Artemia* is a non-selective filter feeder capable of filtering particles between 1 and 50 μm [8], which allows it to serve as a live carrier for encapsulating and transporting specific nutrients within its digestive tract [9]. Although previous research has primarily focused on enriching *Artemia* with essential polyunsaturated fatty acids (PUFAs), phospholipids, amino acids, and fat-soluble vitamins to meet larval nutritional requirements [10], the use of *Artemia* as carotenoid carriers in the aquaculture food chain is gaining increasing attention [11].

Similar to most crustaceans, *Artemia* cannot synthesize carotenoids and must acquire them from their diets. While endogenous carotenoids are present in *Artemia* eggs and newly hatched nauplii, their levels decline significantly during as development progresses in the absence of exogenous carotenoid supplementation [12,13]. Carotenoids from microalgae, serving as a primary source of natural feed, can be efficiently transferred to *Artemia*, thereby enhancing the growth and health of metanauplii and adults through carotenoid accumulation [9,14]. Although there has been substantial research on the biochemical pathways of carotenoid metabolism in *Artemia,* studies focused on the underlying genes and transcriptomic data remain limited. RNA sequencing (RNA-seq) has emerged as a cost-effective strategy for exploring carotenoid metabolism-related genes on a genome-wide scale [15]. This study provides fundamental data on qualitative and quantitative changes in carotenoids within *Artemia*, with and without carotenoid enrichment, and offers insights into the molecular mechanisms underlying carotenoid metabolism in crustaceans.

## 2. Materials and Methods

### 2.1. Artemia Cyst Incubation

*Artemia* metanauplii were obtained by hatching *Artemia sinica* cysts. The cysts were initially decapsulated with sodium hypochlorite (2 g of *Artemia* cysts/1 g of active chlorine) and subsequently neutralized with sodium thiosulfate (Na_2_S_2_O_3_) dissolved in filtered seawater (2%, *w/v*). Following this treatment, the cysts were incubated in a 10 L cylindro-conical PVC container at a density of 2 g/L filtered rearing seawater (salinity: 30 ‰; temperature: 28 °C; pH: 8–8.5) with continuous aeration and 2000 lux illumination over 24 h, in accordance with the protocol of Reis et al. [7]. After hatching, nauplii were separated from hatching debris and transferred to a 20 L cylindrical PVC container with fresh rearing seawater, where they were held until they reached the metanauplius stage (Instars II through V, mouth and anus opening). At this stage, the desired number of metanauplii were randomly selected for subsequent enrichment and starvation procedures.

### 2.2. Artemia Enrichment Experiment Design

During the enrichment process, *Artemia* metanauplii were maintained under the previously described experimental conditions, with the diet supplemented with a pure carotenoid emulsion. This emulsion was prepared by dissolving 0 mg (control), 100 mg, 300 mg, or 500 mg of synthesized β-carotene (98% purity, Adamas) in a minimal amount of chloroform, which was subsequently mixed with Tween 80. Following thorough mixing, the chloroform was removed from the carotenoid emulsions using a nitrogen blower (HUXI, Shanghai, China).

The enrichment procedure involved incubating *Artemia* metanauplii (30 individuals/mL) with β-carotene substrates for 24 h in sixteen 30 L cylindrical PVC containers, each containing 20 L of filter seawater. Four experimental enrichment treatments (0 mg/L, 5 mg/L, 15 mg/L, and 25 mg/L β-carotene) were established using the prepared carotenoid emulsions, with each treatment replicated four times. To examine carotenoid dynamics, metanauplius samples were collected after 6, 12, 18, or 24 h of exposure, then stored at −80 °C for subsequent analyses.

### 2.3. Artemia Starvation Experimental Sampling After β-Carotene Enrichment

An independent experiment was conducted to investigate the carotenoid metabolic conversion in *Artemia* metanauplii following β-carotene enrichment. After the 12 h enrichment procedure described above, enriched *Artemia* were transferred to sixteen fresh 30 L cylindrical PVC containers with non-pigmented rearing seawater and were fed appropriate quantities of pigment-free yeast for an additional 24 h. Samples of *Artemia* were collected every 6 h and stored for further analysis.

### 2.4. Analysis of the Carotenoid Content and Composition

**Figure 1:**
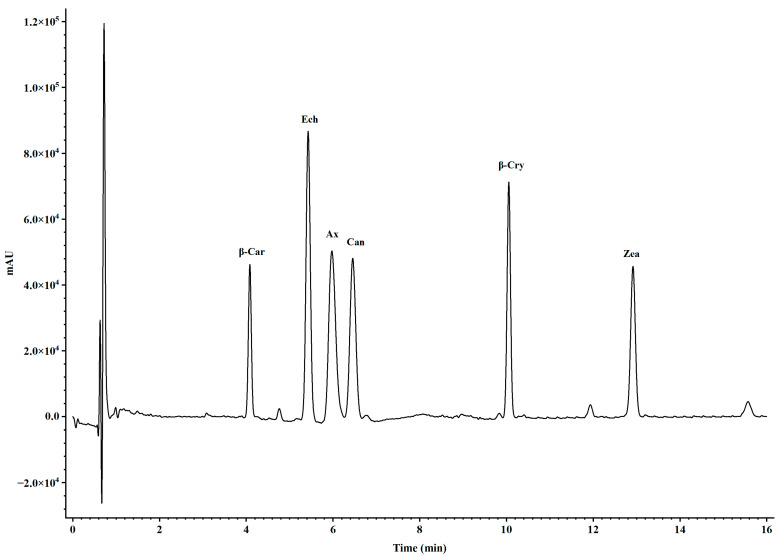
Peak curves of carotenoid standards detected by UPC^2^-PDA with gradient elution conditions. The notations above the peaks represent β-car, β-carotene; Ech, echinenone; Ax, astaxanthin; β-Cry, β-cryptoxanthin; and Zea, zeaxanthin.

Carotenoids were extracted from freeze-dried *Artemia* metanauplii using an acetone and methanol solution (2:1, *v/v*). The extraction process was repeated at least three times until no further color was observed in the solvent. The resulting extracts were pooled into new tubes and subsequently dried using a nitrogen blower. The carotenoid residues in each tube were redissolved in a mobile phase solution (acetonitrile/methanol, 70:30, *v/v*) and filtered through 0.22 μm hydrophilic polypropylene discs (Pall Corp) for the carotenoid composition analysis. All extraction procedures were conducted under low light conditions to minimize the photo-oxidation of the carotenoids. For the compositional analysis, samples were analyzed via ultraperformance convergence chromatography (UPC^2^, Waters ACQUITY, Milford, CT, USA) using a Waters ACQUITY HSS C_18_ SB column (1.8 μm, 2.1 mm × 150 mm). The UPC^2^ conditions included a mobile phase consisting of A (100% liquid CO_2_) and B (100% methanol) with a flow rate of 0.6 mL/min and an injection volume of 10 μL. The gradient program began with an initial ratio of 90% A to 10% B for 7 min, following by a shift to 80% B to 20% C for 5 min, and a return to the initial ratio over 4 min. Carotenoid concentrations in each sample were detected at 475 nm using a photodiode array (PDA) detector and quantified by calculating peak areas against known standards, including astaxanthin, zeaxanthin, β-cryptoxanthin, canthaxanthin, echinenone, and β-carotene (Figure 1). Standards for astaxanthin, canthaxanthin, zeaxanthin, echinenone, and β-carotene were obtained from Sigma-Aldrich (St. Louis, MO, USA), while the β-cryptoxanthin standard was sourced from CaroteNature (Zurich, Switzerland).

### 2.5. Extraction and Sequencing of RNA from Artemia

Total RNA was extracted from *Artemia* metanauplii after the 12 h enrichment procedure using RNAiso Plus reagent (Takara, Osaka, Japan) following the manufacturer’s protocol. Genomic DNA was subsequently removed using the RapidOut DNA Removal kit (Thermo, Waltham, MA, USA). The RNA quality and concentration were assessed using 1.5% agarose gel electrophoresis (Thermo, Waltham, MA, USA) and quantified with a NanoDrop 2000 microvolume spectrophotometer (Thermo, Waltham, MA, USA). The RNA quality number (RQN) was further evaluated using an Agilent 2100 Bioanalyzer (Agilent, Santa Clara, CA, USA), with only high-quality RNA (RQN > 6.5) proceeding to cDNA library construction. The cDNA library was prepared using the Illumina TruSeq RNA Sample Prep Kit (Illumina, San Diego, CA, USA), following the manufacturer’s instructions, by enriching mRNA with immobilized oligo(dT) primers. The mRNA was fragmented using fragmentation buffer and subsequently reverse transcribed into cDNA with random hexamers. The cDNA libraries were sequenced on the Illumina NovaSeq 6000 platform by Majorbio Co., Ltd. (Shanghai, China).

### 2.6. De Novo Assembly

To obtain high-quality clean reads for the de novo assembly, raw RNA-seq reads were filtered by removing those containing: (1) adapter sequences, (2) more than 10% ambiguous “N” bases, and (3) over 40% of low-quality bases (Q < 20). Clean reads were de novo assembled using Trinity version 2.4.0 [16] To increase the assembly quality, all the assembled sequences were filtered by CD-HIT 4.8.1 [17] to obtain the unigenes. Annotations for the assembled unigenes were conducted via BLAST against multiple databases, including GO, KEGG, eggNog, NR, SwissProt, and Pfam. Differentially expressed genes (DEGs) were identified using DeSeq2 with thresholds of a log2 fold change ≥1 and *p* < 0.05. An enrichment analysis of DEGs (control vs. EG) was conducted using GO terms and KEGG pathways, with the results visualized in bubble plots and histogram generated by GraphPad Prism 9.0. Principal component analysis (PCA) was also conducted to assess similarities and differences in the expression profiles of all samples.

### 2.7. Validation of the Transcription Data with qRT-PCR

Thirteen DEGs were randomly selected for qRT-PCR validation to assess the reliability of the transcriptome data. All primers (Table 1) were designed using Primer-BLAST online software (https://www.ncbi.nlm.nih.gov/tools/primer-blast/index.cgi?LINK_LOC=BlastHome, accessed on 1 August 2023) The reactions were conducted using an Agilent AriaMX system, with β-actin serving as the internal reference gene. The reaction mixture consisted of 0.8 μL of the forward primer (10 μM), 0.8 μL of the reverse primer (10 μM), 3 μL of the cDNA template, and 10 μL of TB Green Premix *Ex Taq* II (Takara, Osaka, Japan), for a total reaction volume of 20 μL. The thermal cycling conditions were as follows: initial denaturation at 95 °C for 30 s, followed by 40 cycles of 95 °C for 5 s and 58 °C for 30 s. The relative expression levels were calculated using the 2^−ΔΔCt^ method.

### 2.8. Statistical Analysis

The results from the current study are reported as means ± standard errors of the means (S.E.Ms., n = 3). The data were subjected to one-way ANOVA using SPSS 20.0 software. Duncan’s multiple range tests were used as post hoc tests. Differences among the treatments’ means were considered significant at *p* < 0.05.

### 2.9. Ethics Statement

The present experimental procedures were carried out in strict accordance with the recommendations in the ethical guidelines of EU Directive 2010/63/EU for animal experiments.

## 3. Results

### 3.1. Change in the β-Carotene Contents of Artemia During the Enrichment and Starvation Processes

**Figure 2:**
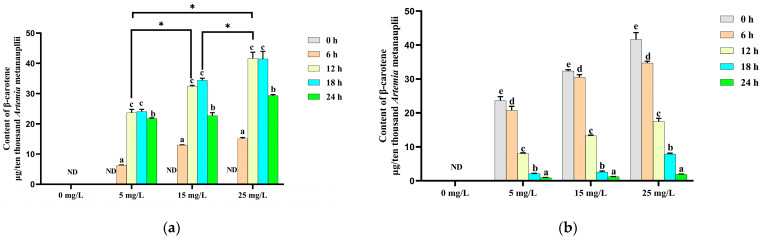
Changes in the β-carotene content during the β-carotene enrichment and starvation processes. (**a**) For the *Artemia* metanauplius enrichment process; (**b**) for the *Artemia* metanauplius starvation process. Data are expressed as the means ± S.E.Ms. from triplicate samples from each group. Bars with different letters represent significant differences between various treatments (*p* < 0.05). The asterisk above the black lines denotes significant differences (*p* < 0.05) between different enrichment groups at 12 h. ND means not detected.

Changes in the β-carotene contents of *Artemia* metanauplii during the enrichment process are presented in Figure 2a. No β-carotene was detected in metanauplii without/prior to β-carotene enrichment. Over a 24 h period, the β-carotene content in enriched metanauplii was significantly increased (*p* < 0.05) across the three levels of β-carotene suspensions. The highest β-carotene content (*p* < 0.05) was observed at the 12th h of enrichment, which remained elevated until the 18th h compared to other time points. Based on these results, β-carotene depletion in metanauplii began after the 12 h enrichment period (Figure 2b). In the absence of further β-carotene intake, β-carotene levels declined significantly (*p* < 0.05) over time in the enriched treatment groups.

### 3.2. Changes in the Endogenous Carotenoid Composition in Artemia During the β-Carotene Enrichment and Starvation Processes

**Figure 3:**
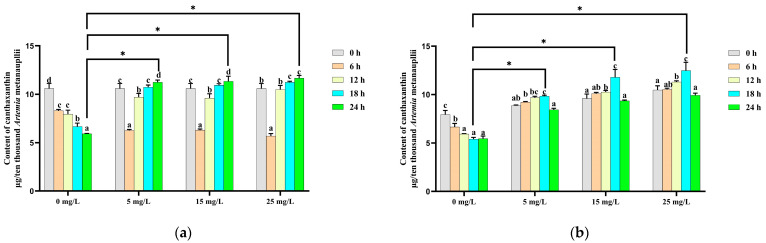
Change in the canthaxanthin content during the β-carotene enrichment and starvation processes. (**a**) For the *Artemia* metanauplius enrichment process; (**b**) for the *Artemia* metanauplius starvation process. Data are expressed as the means ± S.E.Ms. from triplicate samples from each group. Bars with different letters represent significant differences between various treatments (*p* < 0.05). The asterisk above the black lines denotes significant differences (*p* < 0.05) between different enrichment groups.

In addition to β-carotene, only canthaxanthin and echinenone were detected in *Artemia* metanauplii before and after the enrichment process in this study. In the control group, the canthaxanthin content decreased significantly (*p* < 0.05) over time in the absence of a carotenoid supplement. In contrast, in the β-carotene-enriched treatment groups, canthaxanthin levels initially declined and then significantly increased with time. The highest canthaxanthin levels in each enrichment treatment group were observed at 24 h and were significantly higher (*p* < 0.05) than in the control group (Figure 3a). During the depletion phase, the canthaxanthin content continued to increase until to 18 h, showing a significantly difference (*p* < 0.05) from the control group at this time point (Figure 3b).

**Figure 4:**
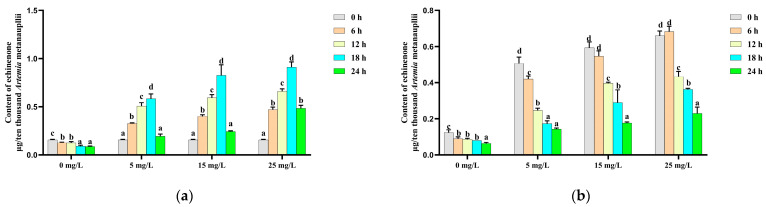
Change in the echinenone content during the β-carotene enrichment and starvation processes. (**a**) For the *Artemia* metanauplius enrichment process; (**b**) for the *Artemia* metanauplius starvation process. Data are expressed as the means ± S.E.Ms. from triplicate samples from each group. Bars with different letters represent significant differences between various treatments (*p* < 0.05).

The echinenone content in the control group exhibited a similar trend to that of canthaxanthin, decreasing steadily over time. In the enriched treatment groups, the echinenone content was significantly increased during the initial 18 h β-carotene enrichment, followed by a significant decline thereafter (Figure 4a). During the depletion phase, echinenone levels in both the control and enriched treatment groups decreased significantly (*p* < 0.05) over time (Figure 4b).

### 3.3. De Novo Sequencing and Assembly

**Figure 5:**
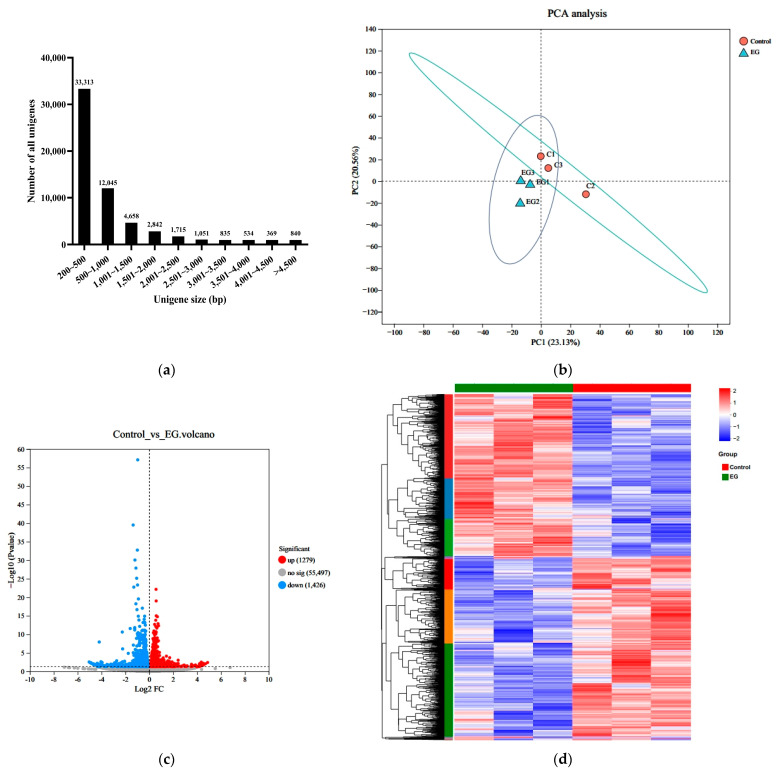
Transcriptome analysis of the *Artemia* metanauplius samples from the treated and control groups. (**a**) Distribution of unigenes with different sizes; (**b**) principal component analysis (PCA) of the *Artemia* metanauplius samples; and (**c**) volcano plot of DEGs. The blue dots indicate that the genes are downregulated. The red dots indicate that the genes are upregulated. The gray dots indicate that there are no significant differences in expression. Each dot represents a gene. (**d**) Heatmap of DEGs among these groups.

Six cDNA libraries were constructed using whole-body *Artemia* metanauplii from two groups: control and EG. Sequencing on the Illumina Novaseq 6000 sequencing platform yielded 51,061,208, 45,635,052, and 46,527,368 raw reads for the control group, and 54,199,590, 45,287,112, and 47,207,506 raw reads were generated for the EG group. After filtering, 50,706,124, 45,299,184, and 46,208,168 clean reads were obtained for the control group, and 53,788,384, 44,898,224, and 46,891,296 clean reads for the EG group. The Q30 quality score for each sample exceeded 90%, indicating high sequencing quality. Following the merging of reads from all samples for assembly, a total of 58,202 unigenes were obtained, with minimum, maximum, and average lengths of 201 bp, 18,835 bp, and 815 bp, respectively, and an N50 values of 1428 bp. The distribution of unigene lengths is presented in Figure 5a.

The assembled unigenes were annotated using BLAST searches of six public databases: Nr (10,203), eggNOG (15,338), GO (9449), KEGG (11,144), Pfam (15,256), and Swiss-Prot (13,401, 23.46%).

### 3.4. Gene Expression and Functional Enrichment Analysis of DEGs

The principal component analysis (PCA) results indicated a clear distinction between the EG group and the control group (Figure 5b). DEGs were identified by comparing the treated and control libraries, focusing on genes with significant changes in expression during the enrichment process. A total of 2705 DEGs, with a false discovery rate (FDR) < 0.05 and |log2 fold change| > 1, exhibited differential transcription. As shown in Figure 5c,d, β-carotene enrichment resulted in 1279 upregulated and 1426 downregulated genes.

**Figure 6:**
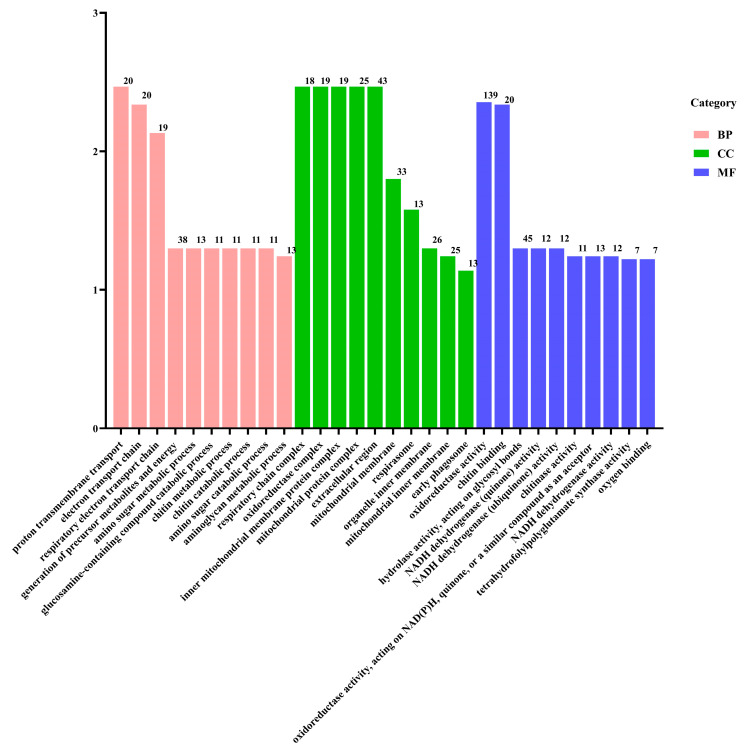
GO classification of the DEGs.

**Figure 7:**
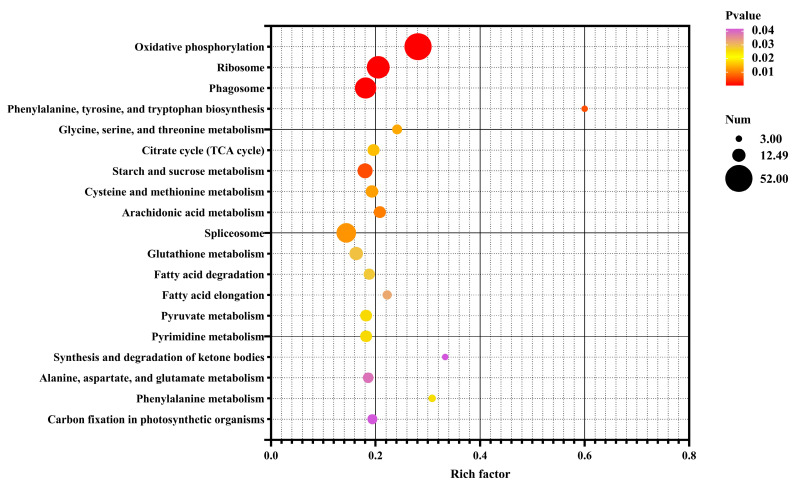
Bubble chart of enriched KEGG pathways for DEGs in the control vs. EG groups. Rich factor is the ratio of the number of DEGs for a certain KEGG pathway to the total number of genes in that pathway. The significance of identified KEGG pathways was determined as *p* < 0.05.

To explore the functional implications of these DEGs, GO and KEGG pathway analyses were conducted. The DEGs were categorized into GO terms, including biological processes (BPs), cellular components (CCs), and molecular functions (MFs). The top 30 enriched GO terms in each category are illustrated in Figure 6. In the BP category, “transmembrane transport” and “electron transport chain” were the most significantly enriched terms. In the CC category, the terms “respiratory chain”, “oxidoreductase”, and “mitochondrial protein” were the most enriched. For the MF category, “oxidoreductase activity” and “chitin binding” were the most significant terms. In the KEGG enrichment analysis, the top three pathways were “oxidative phosphorylation”, “ribosome”, and “phagosome”, which are crucial for cellular metabolic processes and homeostasis. Furthermore, the DEGs were predominantly enriched in metabolic pathways associated with the regulation of energy production (Figure 7).

### 3.5. Validation by qRT-PCR

**Figure 8:**
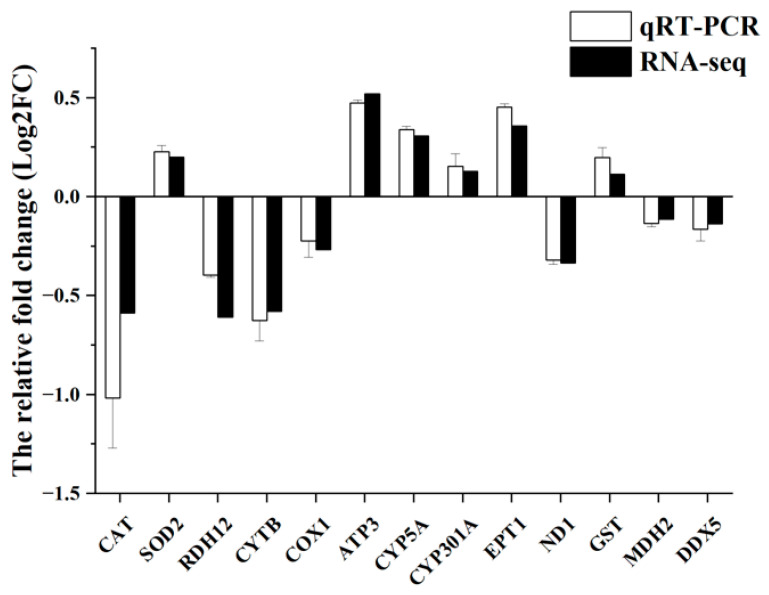
Validation of RNA-seq data by qRT-PCR. qRT-PCR data are shown as the means ± S.E.Ms. (n = 3) from three biological replicates utilized in RNA-seq.

To validate the RNA-seq analysis results, thirteen DEGs were randomly selected for validation using qRT-PCR. As shown in Figure 8, the expression patterns obtained from qRT-PCR closely matched those derived from RNA-seq data, indicating the reliability of the sequencing results and confirming the accuracy of the transcriptome analysis employed in this study.

## 4. Discussion

### 4.1. Changes in the Carotenoid Content and Composition in Artemia in Response to β-Carotene Enrichment

Carotenoids in crustaceans are broadly categorized into two types: carotenes and xanthophylls [2]. Among carotenes, β-carotene is the most prominent, while xanthophylls are oxygenated derivatives of carotenes, primarily including β-cryptoxanthin, zeaxanthin, echinenone, canthaxanthin, and astaxanthin [18,19]. The composition and content of carotenoids in crustaceans vary significantly across species, strains, ages, body weights, and physiological stages [20,21], and are closely linked to environmental conditions and dietary factors [22]. Carotenoids in *Artemia salina* have been isolated and analyzed extensively. Echinenone and canthaxanthin, in a ratio of 19:1, are the only carotenoids present in California *Artemia* cysts and newly hatched nauplii [23]. From the metanauplius to adult stages, significant carotenoid accumulation occurs in *Artemia*, primarily derived from the assimilation of carotenoids synthesized by microorganisms within the food chain [13,24]. Consistent with previous findings, the current research demonstrates that, in the absence of exogenous carotenoid intake, environmental factors do not induce the production of xanthophylls beyond canthaxanthin and echinenone throughout the *Artemia* life cycle [25]. In this study, a method for feeding pure β-carotenoid to carotenoid-depleted *Artemia* metanauplii is presented. Additionally, feeding β-carotenoid-rich microalgae (*Dunaliella salina*) to *Artemia* metanauplii effectively enhanced the carotenoid content [9]. These findings confirm that *Artemia* metanauplii and adults can act as bio-encapsulated carotenoid carriers, offering a natural carotenoid source for seed breeding and broodstock [6,14]. Furthermore, *Artemia* exhibit efficient hydrolysis of fatty acid-esterified forms into free compounds, particularly from algal and plant-derived dietary carotenoids, thereby improving the bioavailability and absorption of these compounds by predators [14,25].

In crustaceans, a natural metabolic pathway exists whereby structurally similar carotenoids can be interconverted through a series of ketonization or hydroxylation reactions on their ionone rings [2,4]. Astaxanthin is generally regarded as the terminal product of this metabolic conversion pathway, with the transformation from β-carotene to astaxanthin represents the key metabolic axis in *Penaeus monodon* [2]. However, the carotenoid composition kinetics observed in the current study indicated that β-carotene in *Artemia* can only be converted into echinenone (4-keto-β-carotene), with canthaxanthin (4,4′-diketo-β-carotene) as the final product. This provides a theoretical foundation for understanding why *Artemia* cannot metabolize β-carotene into astaxanthin, likely due to the absence of hydroxylase enzymes required for this pathway. Additionally, dietary precursors such as zeaxanthin and lutein do not yield astaxanthin in *Artemia*, further underscoring the substrate specificity of the β-carotene ketolase in this species [25]. Given the ease of preservation and hatching of *Artemia* cysts, establishing a metabolic model for the conversion of β-carotene to canthaxanthin in *Artemia* could provide valuable insights into carotenoid metabolic mechanisms in crustaceans. Previous studies have also documented geometric and structural isomers of canthaxanthin, with their relative proportions varying significantly at different developmental stages [13]. The selective accumulation of carotenoids, including distinct isomers with sub-molecular structural variations, in various crustacean tissues suggests that these structural differences may have specific physiological effects on the growth, development, and health of aquatic organisms. This finding also underscores the complexity of interactions between carotenoids and other biomolecules.

### 4.2. Genes Associated with Carotenoid Physiological Function

Carotenoids play essential nutritional and physiological roles in crustaceans, impacting not only pigmentation but also promoting growth, enhancing immunity, and improving reproductive performance and stress resistance [4,26]. Supplementation with carotenoids in feed has been shown to significantly enhance growth performance and survival rates in crustaceans [27]. Additionally, carotenoids have been reported to positively influence survival and metamorphosis during the larval stages [2,28]. Studies further indicates that carotenoid intake is often positively correlated with pigment deposition and growth performance in crustaceans [29]. Moreover, compared to fatty acids, carotenoids contribute more significantly to the growth and health of *Artemia*, leading to improvements in the survival rate, average body length, total effective body length, and total antioxidant capacity [9]. Consistent with these findings, metabolic pathways related to growth, such as amino acid metabolism and ketone body metabolism, were significantly upregulated, suggesting that dietary carotenoids may positively impact *Artemia* growth. Research has shown that lipid transport proteins, rather than specific carotenoid-binding proteins, are primarily involved in carotenoid absorption, secretion, and intracellular transport [30]. Consequently, the KEGG enrichment analysis revealed upregulated pathways related to lipid metabolism, including fatty acids, sphingolipids, glycerides, and fatty acid biosynthesis, which aligns with the requirement of lipid transport proteins for carotenoid transfer. Additionally, the significant upregulation of the “ribosome” and “oxidative phosphorylation” pathways suggests that carotenoid metabolism demands substantial amounts of energy and protein, supporting the hypothesis that the carotenoid concentration reflects mitochondrial function [15]. Carotenoids are widely used as supplements in aquaculture due to their antioxidant and anti-inflammatory properties [31]. Glutathione, a crucial antioxidant, plays an fundamental role in protecting cells from oxidative damage [32]. The *gsto1* gene, which is essential for glutathione biosynthesis, was significantly upregulated among the DEGs. Other antioxidant-related genes, such as *gpx*, also showed significant upregulation, consistent with previous studies [33]. Moreover, several genes involved in ferroptosis and the MAPK signaling pathway, such as *fgh1* and *hsp90*, exhibited significant upregulation. Ferroptosis, a form of regulated cell death caused by the accumulation of lipid peroxides within cellular membrane, is closely associated with growth, oxidative stress, and immunity [30,34].

### 4.3. Genes Associated with Carotenoid Absorption and Metabolism

Carotenoids, as large organic molecules, are absorbed in aquatic animals through mechanisms similar to those used for lipid absorption [2]. Digestive enzymes facilitate the release of free carotenoids from protein-bound and esterified forms, enabling them to form chylomicrons with other lipids and bile salts, which enhances their bioavailability [35]. Carotenoids are then absorbed by enterocytes at the brush border of the intestinal mucosa, either through passive diffusion or active transport across the membrane bilayer, where they perform various physiological functions [36]. The remaining carotenoids are temporarily stored in various tissues and organs, such as the liver, in the form of free carotenoids, monoesters, diesters, or protein complexes, and are transported via the lymphatic and circulatory systems [37]. Depending on the intake of exogenous carotenoids, free carotenoids can be hydrolyzed and released to maintain a dynamic balance of carotenoid levels across different tissues, thereby supporting normal cellular functions [2].

The efficiency of carotenoid absorption is regulated by various lipid transport proteins rather than by any single specific protein [38]. Three main receptors are believed to facilitate carotenoid transmembrane transport [39]. Scavenger receptor class B1 (*Scarb1*) is a key mediator of carotenoid-based coloration in both vertebrates and invertebrates [40]. However, in the present study, no significant differences in *scarb1* expression levels were observed between the two groups. Cluster determinant 36 (CD36), also known as fatty acid transporter, is implicated in the cellular uptake of β-carotene [41]. A significant up-regulation of *cd36* was observed in the treated libraries compared to the control library in this study. Additionally, several ATP-binding cassette transporters (ABC), recognized for their roles in lipid transport, have been linked to carotenoid transport in previous studies [42]. The significant upregulation of *Abca1* and *Abcb1* in the β-carotene-enriched group suggests that ABC transporters may also contribute to carotenoid transport in *Artemia*, similar to observations in *Crassostrea gigas* [43].

Carotenoids can be classified into two categories based on function: provitamin A carotenoids (such as β-carotene and cryptoxanthin) and non-provitamin A carotenoids (such as lutein, canthaxanthin, and zeaxanthin). Among these, β-carotene exhibits 100% provitamin A activity and serves as the primary source of vitamin A [2]. In animals, carotenoids undergo symmetric and asymmetric cleavage by enzymes to produce vitamin A and its derivatives. β-Carotene-15, 15′-dioxygenase (BCO1) is an intracellular enzyme with high substrate specificity, exclusively catalyzing carotenoids that act as vitamin A precursors [44]. However, *BCO1* was not detected in our transcriptome data. In contrast, β-carotene-9, 10-oxygenase (BCO2) is a mitochondrial enzyme with broader substrate specificity and is capable of metabolizing non-vitamin A precursor carotenoids and participating in biological processes beyond vitamin A synthesis [45]. *BCO2* has been significantly associated with fish flesh pigmentation and carotenoid accumulation [46]. Nonetheless, we observed no differences in *BCO2* expression levels between the control and β-carotene-enriched groups, aligning with the enzyme’s established role in promoting white pigmentation in various animals.

The functional roles of ketolase and hydroxylase are critical in regulating carotenoid metabolic conversion [47]. Research on the key enzyme genes and mechanisms involved in the conversion pathway from β-carotene to astaxanthin has been relatively systematic and comprehensive, though primarily limited to microalgae, bacteria, and fungi capable of synthesizing astaxanthin autonomously [48,49]. In *Haematococcus pluvialis*, which shares an astaxanthin biosynthesis pathway similar to that of crustaceans, β-carotene is converted into astaxanthin through the hydroxylation of the 3,3′ positions of the β-ionone rings catalyzed by β-carotene hydroxylase encoded by the *Crt-b* gene, and the addition of two keto groups at the 4,4′ positions by β-carotene ketolase encoded by the *bkt* gene [50]. Further studies have identified multiple types of *bkt* genes (bkt1, bkt2, and bkt3), where gene duplications add specificity, complexity, and flexibility to the carotenoid metabolic pathway [51]. Recent research has implicated cytochrome P450 family members, *CYP2J19* and *CYP3A80*, as ketolases responsible for keto-carotenoid formation in animals [52]. Although these two genes were not present among the DEGs in this study, several P450 family genes exhibited varied expression profiles. Research on carotenoid metabolism in crustaceans remains limited; however, a bioinformatic analysis of high-throughput transcriptome data in copepods (*Acartia fossae*) has identified differentially expressed β-carotene hydroxylase genes involved in metabolic conversion pathways [53]. Nevertheless, the functional mechanisms of these genes remain unexplored. The absorption, metabolism, transport, and deposition of carotenoids in crustaceans are highly complex processes, with many aspects yet to be fully elucidated. Further investigations are necessary to clarify the detailed mechanisms of carotenoid esterification and hydrolysis, the specific metabolic pathways of carotenoid transformation, the cellular mechanisms of carotenoid uptake, and the genetic regulation underlying carotenoid transport and deposition. A more comprehensive understanding of these processes could facilitate the optimization of feed formulations and feeding strategies, ultimately enhancing the growth, health, and nutritional quality of carotenoid-rich crustaceans in aquaculture.

In conclusion, the observed increases in the β-carotene, canthaxanthin, and echinenone contents during enrichment, along with the depletion dynamics during starvation, underscore *Artemia*’s potential as a bio-encapsulated carotenoid source in aquaculture. Furthermore, the gene expression analysis illuminates the intricate metabolic pathways and gene regulations associated with carotenoid transport, absorption, and conversion, indicating potential avenues for further research on carotenoid metabolism in crustaceans.

## Figures and Tables

**Table 1:** Target gene primers used for qRT-PCR. The gene profiles were normalized that of the β-actin *gene*.

Gene Name	Description	Primers
CAT	catalase	F: CTCCGCTTTCTTGCTCATCT R: CAGCCAGCCTTAGTCTTTCA
RDH12	protochlorophyllide reductase	F: CCCGAGTCAGGAATTGGCTT R: GCATCAGAGGCCTTGTAGCA
COX1	cytochrome c oxidase subunit 1	F: TACTGTGGGAATGGACGTTGA R: CGTGTCCCGTGTAAAGTTCC
SOD2	superoxide dismutase 2	F: CACAAAAGAATCACAAGCAGTAAGG R: TCAAGGCTCAGGATGGGGA
ATP3	ATP synthase subunit gamma	F: GTGTCTATTCGCCTGAAGTCTGT R: GCTTTGGTATATTTTGCTGCTG
CYTB	cytochrome b, partial	F: AGCGGCCATCACAAGAAACA R: AACTACGGTTGGCTGCTCC
EPT1	choline/ethanolamine phosphotransferase 1	F: CTCGTCTATGATTGGACCCTTG R: CAAAGCCAAAGAACGTAATACTCC
MDH2	malate dehydrogenase	F: TCCAAACAGGATTTTCGGTG R: AAAAGAAACAGATGGCGTGC
ND1	NADH dehydrogenase subunit 1	F: TCCTACAACCTTTTTCTGATGGG R: GGAGCAACGAGGTACGGTATG
GST	glutathione transferase family protein	F: TAGCCAGCAAAGTAAAATCAGC R: AGGTGCCACTTCTCTTGACG
DDX5	p68DDX5 RNA helicase	F: CGCTCTTGCTGCTGCTTGT R: GGAGGAGAAATTAAACCAGTTGC
cyp301a	hypothetical protein SPRG_05930	F: AATCATCCCCTTTTAGCCACC R: GCTATCACCTGCCGTTTTTG
cyp5a	cytochrome P450	F: TTGGTTCAACTCGTGCTGCG R: GGAGCTGGCCCAAGGAATTG
β-actin	actin	F: GGTCGTGACTTGACGGACTATCT R: AGCGGTTGCCATTTCTTGTT

## Data Availability

Data are contained within the article.
